# Dr. J. H. Tilden

**Published:** 1902-07

**Authors:** 


Dr. J. H. Tilden, of Buffalo, died suddenly at his home 198 Franklin Street, June 14, 1902, aged 74 years. Dr. Tilden graduated in medicine in 1850, and at one time served as assistant surgeon of the 74th regiment, New York State National Guard. In 1859, owing to his father's ill health he joined him in business and after the former's death continued to conduct the business of contracting and building in his own name. He has constructed some of the largest buildings in Buffalo, including several churches, theatres, hospitals and private residences. He was a member of the Buffalo Historical Society and of several other organisations. Two sons, George T. M. and Hyde F. Tilden, and two daughters, Mrs. H. J. Coleman and Miss Charlotte Tilden, survive. His wife died a few years ago.

